# Ultra-Processed Food Consumption is Associated with Renal Function Decline in Older Adults: A Prospective Cohort Study

**DOI:** 10.3390/nu13020428

**Published:** 2021-01-28

**Authors:** Jimena Rey-García, Carolina Donat-Vargas, Helena Sandoval-Insausti, Ana Bayan-Bravo, Belén Moreno-Franco, José Ramón Banegas, Fernando Rodríguez-Artalejo, Pilar Guallar-Castillón

**Affiliations:** 1 Department of Preventive Medicine and Public Health, School of Medicine, Universidad Autónoma de Madrid-IdiPaz, CIBERESP (CIBER of Epidemiology and Public Health), 28029 Madrid, Spain; jimena.reygarcia@gmail.com (J.R.-G.); helenagabar@gmail.com (H.S.-I.); joseramon.banegas@uam.es (J.R.B.); fernando.artalejo@uam.es (F.R.-A.); mpilar.guallar@uam.es (P.G.-C.); 2 Internal Medicine Department, Ramón y Cajal University Hospital, 28034 Madrid, Spain; 3 IMDEA-Food Institute, CEI UAM+CSIC, 28049 Madrid, Spain; 4 Unit of Nutritional Epidemiology, Institute of Environmental Medicine, Karolinska Institutet, 171 77 Stockholm, Sweden; 5 Department of Nutrition, Harvard T.H. Chan School of Public Health, Boston, MA 02115, USA; 6 Department of Nutrition, 12 de Octubre Hospital, 28041 Madrid, Spain; a.bayan.bravo@gmail.com; 7 Department of Microbiology, Radiology, Pediatrics and Public Health, Universidad de Zaragoza, 50009 Zaragoza, Spain; mbmoreno@posta.unizar.es; 8 Instituto de Investigación Sanitaria Aragón, Hospital Universitario Miguel Servet, 50009 Zaragoza, Spain

**Keywords:** ultra-processed food, creatinine serum levels, glomerular filtration rate, renal function decline

## Abstract

Ultra-processed food (UPF) consumption has been associated with increased risk of cardiovascular risk factors and mortality. However, little is known on the UPF effect on renal function. The aim of this study is to assess prospectively the association between consumption of UPF and renal function decline. This is a prospective cohort study of 1312 community-dwelling individuals aged 60 and older recruited during 2008–2010 and followed up to December 2015. At baseline, a validated dietary history was obtained. UPF was identified according to NOVA classification. At baseline and at follow-up, serum creatinine (SCr) and estimated glomerular filtration rate (eGFR) levels were ascertained and changes were calculated. A combined end-point of renal decline was considered: SCr increase or eGFR decreased beyond that expected for age. Logistic regression with adjustment for potential confounders was performed. During follow-up, 183 cases of renal function decline occurred. The fully adjusted odds ratios (95% CI) of renal function decline across terciles of percentage of total energy intake from UPF were 1.56 (1.02–2.38) for the second tercile, and 1.74 (1.14–2.66) for the highest tercile; *p*-trend was 0.026. High UPF consumption is independently associated with an increase higher than 50% in the risk of renal function decline in Spanish older adults.

## 1. Introduction

Renal function shows a steady decline when ageing [1]. This decline might be increased under different circumstances (such as the presence of cardiovascular risk factors), even leading to the development of a Chronic Kidney Disease (CKD). [2] CKD affects 10% of the world’s population [3] and ranks in the top ten non-communicable diseases contributing to disability and premature death [4]. CKD is linked to high health care costs, a poor quality of life, serious adverse health outcomes [5,6] such as cardiovascular disease, renal failure requiring replacement therapy, infection, or depression, as well as mortality [3]. Over the last decade, a 41.5% increase in CKD mortality has been observed worldwide [7]. Therefore, the decline in the renal function has substantial clinical and therapeutic consequences among the elderly, as well as public health relevance.

The extent to which age-related renal decline is preventable remains controversial [8]. While dietary changes are one of the key modifiable risk factors for progression of CKD [9], they are also useful in the prevention of obesity, hypertension, and diabetes [10]. In fact, a recent meta-analysis of cohort studies has shown that a high adherence to a healthy dietary pattern (rich in whole grains, vegetables, fruit, legumes, nuts, and fish, while low in red and processed meat, sodium, and sugar sweetened beverages) might prevent CKD [11].

Despite the evidence of the benefits of a healthy diet, there has been a rapid westernization of our diet, including a global increase in the consumption of ultra-processed food (UPF) [12,13]. In some high-income countries, the current consumption of UPF accounts for more than 50% of the total energy intake [14,15]. In Spain, several studies have shown that UPF consumption represents 24% of total energy intake [16], which could be from 1.5 to 5 servings per day [17].

UPF is formulated mostly or entirely with substances derived from food with little, if any, of the original food remaining. Industrial processing also involves the addition of chemicals to improve shelf-life and the organoleptic characteristics of UPF [18]. Compared with other food groups, UPF is typically low-cost, ready to consume, and hyper-palatable [19]. Moreover, UPF is low in fiber and micronutrients, while it is high in refined carbohydrates, added sugars, saturated, and trans fatty acids, sodium, and additives [20,21,22].

Systematic reviews of the literature have shown a positive association between UPF consumption and adverse health outcomes [19,23], including obesity [24,25], diabetes [26], hypertension [27], and mortality [16,17,28,29]. However, to our knowledge, no previous study has evaluated the role of UPF consumption renal function decline. Therefore, this study aimed to prospectively assess the association between UPF consumption and the risk of renal function decline in the Seniors-ENRICA-1 study, which is a population-based cohort of older adults from Spain. We focused specifically on older adults, where UPF consumption is increasing and renal function worsens with age.

## 2. Materials and Methods

### 2.1. Study Design and Participants

Data were collected from the Seniors-ENRICA-1 cohort, which was established during 2008–2010. This is a representative cohort of the non-institutionalized population aged 60 and older in Spain, whose methods have been previously reported [30,31]. At baseline, a computer-assisted phone interview was performed to obtain information on sociodemographic factors, lifestyle, and morbidity. In addition, two subsequent home visits were performed to collect blood and urine samples, to obtain a dietary history, and to conduct a physical examination. Participants were followed-up until 2015, when another wave of data collection was carried out. Participants who were reported dead or declined another interview or were to have a blood sample collected were excluded. Study participants gave written informed consent. The Clinical Research Ethics Committee of La Paz University Hospital in Madrid (Spain) approved the study.

### 2.2. Study Variables

#### 2.2.1. Diet and Covariables

At baseline, information on diet was collected through a validated computerized face-to-face dietary history (DH-ENRICA), developed from that used in the EPIC (European Prospective Investigation into Cancer and Nutrition) cohort study in Spain [32,33]. Participants were asked about the food consumed in a typical week of the preceding year. The HD-ENRICA registers 860 foods and 24 different cooking methods, and uses 120 sets of photographs to help in estimating the portion sizes. Trained and certified interviewers performed the data collection. The intake of macro and micronutrients was estimated using standard food composition tables for Spain.

At baseline, self-reported information was obtained on sex, age, educational level (no formal education or primary, secondary, and university), smoking status (never, former, and current smokers), and former-drinker status. Physical activity was ascertained at baseline with the questionnaire developed by the EPIC group and was expressed in metabolic equivalents (MET)–hour/week [34], and the number of hours watching TV per week was self-reported. Participants reported the following physician-diagnosed chronic conditions: coronary heart disease, heart failure, stroke, chronic respiratory disease, cancer, osteoarthritis, and depression requiring treatment. A nurse checked the number of the reported medications used against drug packages. Hypertension was defined as having a systolic blood pressure ≥140 mmHg or a diastolic blood pressure ≥90 mmHg or antihypertensive treatment. Diabetes was defined as having a fasting glucose ≥126 mg/dl or antidiabetic treatment. Hypercholesterolemia was defined as having a total cholesterol ≥200 mg/dl or drug treatment. Finally, weight and height were measured at home under standardized conditions, and body mass index (BMI) was calculated as weight in kg divided by height in m squared.

##### Exposure Assessment and NOVA Classification

Foods consumed were categorized into four groups according to the NOVA classification based on the extent and purpose of industrial food processing [35,36]. In brief, the first group includes unprocessed or minimally processed food such as fruit and vegetables, grains, nuts and seeds, fresh and pasteurized milk, and natural yogurt with no added sugars or artificial sweeteners. The second group comprises processed culinary ingredients (salt, sugar, honey, vegetable oils, butter, lard, and vinegar). The third group includes processed foods, for example canned or bottled vegetables and legumes, fruit in syrup, canned fish, unpackaged cheeses, freshly made bread, and salted or sugared nuts and seeds. Finally, the fourth group consists of UPF, which are those formulated mostly or entirely from food-derived substances containing little or none of the original food form, for example snacks, cookies, sweets, ice-cream, pizza, instant soup, processed meat, or soft-drinks. The recorded foods as well as their group according to the NOVA classification, has been previously reported in detail [16].

#### 2.2.2. Renal Function Decline

At baseline and at the end of follow-up, 12 h fasting blood was extracted and a spot urine sample was provided during the home visit. Laboratory determinations were performed centrally at the Center of Biological Diagnosis of the Hospital Clinic in Barcelona, using standard procedures and appropriate quality controls [30]. Serum creatinine (SCr) was determined by the Jaffé, alkaline picrate by kinetic reaction. The estimated glomerular filtration rate (eGFR) was estimated from SCr with the Chronic Kidney Disease Epidemiology Collaboration (CKD-EPI) Equation [37]. Changes in SCr and eGFR levels from baseline to the end of follow-up were calculated. Renal function decline was defined as a SCr increased or an eGFR decreased beyond that expected for age. Change in eGFR beyond that expected for age was calculated in 3 steps: (i) eGFR based on baseline creatinine and age in 2015; (ii) eGFR in 2015 based on both SCr and eGFR in 2015; and (iii) subtracting ii from i.

### 2.3. Statistical Analysis

**Figure 1:**
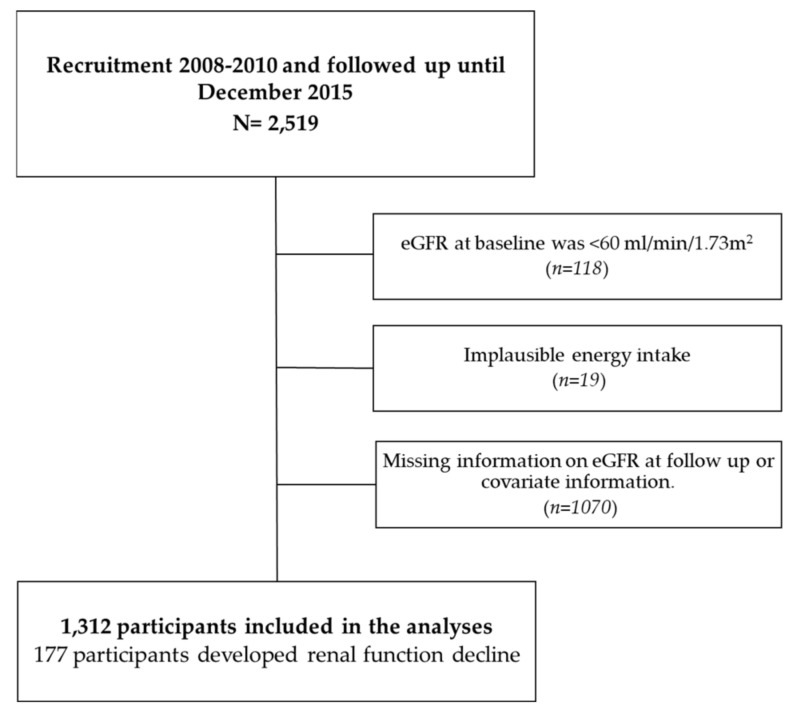
Participants’ flowchart.

The Seniors-ENRICA cohort comprises 2519 participants from the ENRICA (Study on Nutrition and Cardiovascular risk factors in Spain) study, who were 60 or over at baseline, and who provided data in 2015. Of them, 118 were excluded if their eGFR at baseline was <60 mL/min/1.73 m^2^ [38]. These participants were excluded because they could have received advice to avoid UPF consumption. We also excluded 19 participants with extreme data in energy consumption (total energy intake out of range: 600–4200 kcal/day in men or 400–3500 in women). We excluded 1062 participants with missing information on eGFR at follow-up, and 8 participants with missing covariate information. Finally, the study sample consisted of 1312 participants without evidence of renal function impairment (Figure 1).

UPF consumption was expressed as the percentage of energy from UPF to total energy intake, as well as in g/day of UPF per kg of body weight. Logistic regression models were used to assess the association between UPF consumption, in sex-specific tertiles, and renal function decline; results were expressed as odds ratios (OR) and their 95% confidence interval (CI), using the first (lowest) tertile as reference. The p for linear trend was calculated modelling the UPF tertiles as a continuous variable.

We built four logistic regression models with consecutive adjustment levels: model 1 was adjusted for sex, age and total energy intake; model 2 was further adjusted for educational level (no formal education or primary, secondary, and university), smoking status (never, former, current smoker), former-drinker status (yes, no), physical activity (METs-hour/week), time spent watching TV (hour/week), and fiber consumption (g/day); model 3 was further adjusted for the number of chronic conditions, taking into account the following: coronary heart disease, heart failure, stroke, chronic respiratory disease, cancer, osteoarthritis, and depression requiring treatment (continuous), number of medications used per day (continuous), and two well-established renal risk factors: hypertension (yes/no) and diabetes (yes/no); hypercholesterolemia (yes/no), and body mass index (BMI) (continuous).

Statistical significance was set at two-sided *p* < 0.05. The analyses were performed with Stata/SE, version 13.1 (StataCorp, College Station, TX). This article follows the recommendations of the STROBE—Nutritional Epidemiology initiative [39,40,41].

## 3. Results

Among the 1312 participants (51% women; mean age 67 ± 5.5), 183 cases of renal function decline occurred by the end of the 6-year follow-up. The average percentages of energy consumption from UPF for each consecutive tertile of UPF (% of energy) were: 8.6%, 18.7%, and 33.0% in men; and 6.8%, 16.2%, and 29.8% in women (Table 1). Compared with participants in the lowest tertile of UPF consumption those in the highest tertile of consumption had a higher energy intake and BMI, were more frequently ex-drinkers, and performed less physical activity (Table 1).

Participants with a higher baseline UPF consumption were more likely to have a decline in their renal function over the follow-up. Compared with the lowest tertile of the percentage of energy intake from UPF, the fully adjusted OR (95% CI) for renal function decline was 1.56 (1.02–2.38) for the second tertile and 1.74(1.14–2.66) for the highest tertile; *p*-trend 0.023 (Table 2, Model 3). The corresponding values when UPF intake was expressed in g/kg/day were 1.28 (0.85–1.85) and 1.62 (1.06–2.49); *p*-trend 0.043 (Table 2, Model 3).

When stratified analyses were performed, results were similar according to the prevalence of several cardiovascular risk factors (having at least one chronic condition, hypertension, diabetes, hypercholesterolemia, and obesity). However, in individuals with diabetes and without obesity, the association was stronger (Table 3).

**Figure 2:**
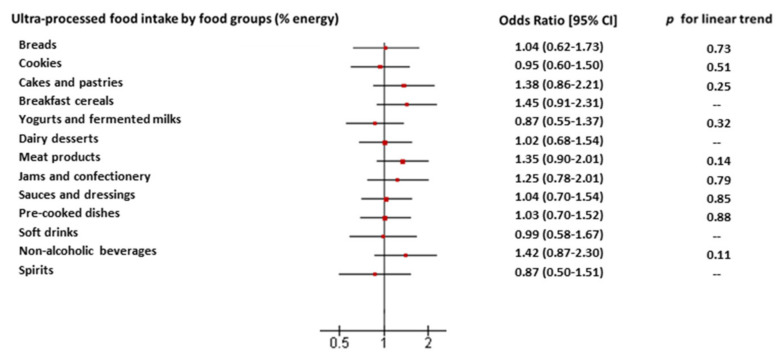
Odds ratio of renal decline risk and 95% confidence intervals (95% CIs) for tertile 3 (highest intake) of groups of ultra-processed food consumption as a percentage of total energy (% of energy) versus tertile 1 (lowest intake), in the Seniors-ENRICA 1 cohort study, *n* = 1312. When the intake of ultra-processed food from a specific food group occurred in less than 25% of the participants, the odds ratio (95% CI) was calculated between subjects who consumed the food compared to those who did not (as in breakfast cereals, dairy desserts, soft drinks and spirits). *p* for linear trend was calculated using tertiles as a continuous variable. Adjustments as in model 3. Other non-alcoholic beverage groups include instant coffee drinks and cocoa drinks, packaged juices and other non-alcohol drinks, excluding soft drinks.

When comparing extreme tertiles of specific UPF food groups, although none of them reached statistical significance, breakfast cereals, non-alcoholic beverages (e.g., industrial fruit juices), cakes and pastries, and meat products, were the ones that contributed the most to this association (Figure 2).

## 4. Discussion

After 6 years of follow-up, UPF consumption was independently associated with renal function decline in a cohort of community-dwelling individuals aged 60 or older from Spain. This study found that participants with the highest UPF consumption at baseline have around a 50% higher risk of renal function decline compared to participants with the lowest consumption after adjusting for a series of demographic, lifestyle as well as clinical and biological covariates. This association could be stronger among diabetics. Our study extends findings from previous studies of dietary patterns and renal impairment risk, and reinforces the importance of diet in the primary prevention of renal decline at a population level.

Some healthy diets have been linked to a lower risk of CKD. A higher adherence to the Mediterranean diet (rich in fruit, vegetables, cereals, legumes, and fish) has been associated with a lower CKD incidence in a multi-ethnic cohort [42]. Similarly, studies of young and middle-aged adults in the United States have found that those with a higher adherence to the Dietary Approaches to Stop Hypertension (DASH) diet (rich in fruit, vegetables, legumes, nuts, and low-fat dairy) also had a lower risk of incident CKD [43]. Another study examining dietary sources of proteins and CKD found that, when one serving of red and processed meat was replaced with plant proteins, the risk of CKD was significantly lower [12]. Likewise, the proportion of CKD attributable risk to a lower adherence to a healthy plant-based diet is estimated at around 4% [44].

By contrast, UPF consumption has previously been associated with hypertension, diabetes, obesity and metabolic syndrome [45], which in turn, are major risk factors for renal function impairment. In addition, in our study UPF consumption increased the risk of renal function decline independently of hypertension, diabetes, BMI, as well as other chronic conditions. Our findings suggest that UPF consumption may be directly associated with an impairment of the renal function.

Several mechanisms may be involved in the results obtained, such as the consumption of a low amount of fiber, as well as high amounts of sodium, sugars, and phosphates, when following a diet high in UPF [14]. In a recent study, fiber intake has shown an inverse association with incident CKD, with a 11% decrease in the risk of CKD for every 5-g increase in fibre intake [46]. Fiber intake also improves glycemic control as well as insulin secretion, which is associated with a lower risk of microalbuminuria and proteinuria [47]. In addition, fiber can reduce the risk of CKD by mitigating the effect of some of their well-established risk factors such as hypertension and diabetes [48].

A higher intake of sodium is also consistently associated with an increased risk of CKD and eGFR decline [11]. The meta-analysis from Bach et al. shows with a moderate quality of evidence that sodium intake was associated with a higher risk of CKD in 6 studies involving 43,772 participants [11]. Likewise, in cohort studies, a diet low in sodium is associated with a lower CKD risk among high-risk individuals. This is the case for the Tehran Lipid and Glucose Study (TLGS) whose participants had dysglycemia, dyslipidemia, and high blood pressure and where a negative association was found between a low-sodium diet (DASH-style diet) and CKD after 3 years of follow-up [49]. In addition, a U-shaped dose-response has been observed between sodium intake and the incidence of CKD, although it could be due to reverse causation when there is low sodium intake. However, even though more research on the optimal level of sodium intake is needed, it is clear that high sodium consumption is detrimental, especially for hypertensive patients [50,51]. Moreover, when CKD is established, there is clinical trial evidence of the detrimental effect of sodium intake on blood pressure and proteinuria [52].

Intake of simple sugars may also play a role. In an analysis of 1630 Iranians from the TLGS, a diet high in fats and sugar was related to a 46% increase in the risk of CKD. Likewise, consumption of >4 servings/week of soft drinks doubled the risk of developing CKD (Yuzbashian et al., 2016) [53]. Similar results were obtained in a community-based cohort of African Americans (the Jackson Heart Study) after 8 years of follow-up (Rebholz, 2020) [54].

Some food additives from UPF, particularly phosphates, deserve a mention [55]. Unlike organic phosphorus, which is present in plant-based food (with low phosphorus availability due to phytate content) [56], inorganic phosphate is present in many UPF as an additive, and has a very high bioavailability [57,58]. This is why inorganic phosphate is disproportionately high in westernized diets compared with organic phosphorus from natural food sources [57]. UPFs that contain high amounts of inorganic phosphates include processed meat, ham, sausages, canned fish, baked goods, cola drinks, and other soft drinks. Individuals with a high consumption of UPF also had a high consumption of added phosphate that could be 250–1000 mg higher than in individuals with a low consumption of UPF [59]. However, dietary phosphate assessment is complex and mostly underestimated, as the amount of food additives containing phosphate is not reported on food labels [57,60].

The rapid consumption of large amounts of phosphate leads to acute kidney injury and ultimately to CKD [55]. Furthermore, large doses of phosphate, as in oral therapy (>2250 mg/day on top of dietary phosphate), over 1–7 years increased the occurrence of calcifications in soft tissues and impaired renal function [61]. High serum phosphate in older adults is also associated with renal dysfunction, cardiovascular risk and premature death [55,62,63].

Phosphate could also operate through other renal risk factors such as hypertension or diabetes [10]. In an 11 week intervention study in young adults with normal renal function, high-phosphate intake was linked to increased pulse rate and systolic and diastolic blood pressure [64]. Likewise, in the French E3N cohort, a higher phosphate intake was associated with a higher incidence of diabetes [65].

The social and health relevance of renal damage is substantial, and its prevention is a public health priority because: (1) CKD imposes a significant economic burden. Many developed countries spend between 2–3% of their annual health care budget to treat the most advanced forms of the disease, for example kidney replacement therapy. (2) Mortality due to renal failure also rose between 2005 and 2017 from 0.9 million to 1.2 million deaths annually [7]. (3) There is a significant potential of prevention (with over 497 million adults worldwide with CKD stages 1–5 [11]). (4) The consumption of UPF continuous to steadily increase globally.

Our study has some limitations. First, as in most nutritional epidemiology studies, diet was self-reported so a plausible recall bias cannot be excluded. Second, certain misclassification of UPF cannot be ruled out either. However, the NOVA classification is easy to apply, and it is the most frequently used in epidemiological studies. Third, the number of people who developed a decline in renal function was small, although the statistical power was enough to find significant associations. Finally, renal decline is based on one creatinine measurement in each time period that may lead to non-differential misclassification and conservative results. This study also had some strengths. These include the prospective design with a relatively long follow-up period, which reduced the possibility of reverse causation. Moreover, diet was collected using a comprehensive and validated dietary history, and analyses were adjusted for the main potential confounders. Finally, this is the first study showing this association.

## 5. Conclusions

In conclusion, in this prospective study of community-dwelling older adults from Spain, a higher intake of UPF was associated with a decline in renal function. These observational findings add evidence for the restriction of the consumption of UPF as a primary prevention strategy for CKD, and the need to promote the consumption of fresh or minimally processed foods over UPF to reduce the burden of disease in the general older population.

## Figures and Tables

**Table 1:** Baseline characteristics of the cohort participants according to sex-specific terciles of Ultra-processed Food Consumption as a percentage of total energy (% energy), in the Seniors-ENRICA Cohort Study (*n* = 1312).

	Ultra-Processed Food Consumption (% energy)			
	T1 (Lowest) (*n* = 438)	T2 (*n* = 438)	T3 (Highest) (*n* = 436)	*p* Trend
Total energy (kcal/day), mean ± SD	1948 ± 549	2053 ± 565	2161 ± 569	<0.001
Ultra-processed food consumption (% energy), mean ± SD	7.7 ± 3.5	17.5 ± 3.0	31.5 ± 7.7	<0.001
Ultra-processed food consumption (grams per day), mean ± SD	128 ± 99	251 ± 141	379 ± 177	<0.001
Weight (kg), mean ± SD	73.7 ± 13	74.8 ± 13	76.0 ± 13.0	0.044
Ultra-processed food consumption (g/kg), mean ± SD	1.8 ± 1.3	3.4 ± 1.9	5.1 ± 2.5	<0.001
Age, years, mean ± SD	67.4 ± 5.5	67 ± 5.2	67 ± 5.8	0.823
Educational level, %				0.735 †
No formal education or primary	23.7	23.9	23.9	
Secondary	25.6	25.8	29.1	
University	50.7	50.2	47.0	
Smoking status, %				0.356 †
Never smoker	57.8	58.9	54.6	
Former smoker	32.2	28.3	32.1	
Current Smoker	10.1	12.8	13.3	
Former-drinker status, %	8.7	4.6	10.8	0.003 †
Physical activity, MET-hour/week, mean ± SD	63 ± 34	60 ± 32	58 ± 34	0.035
Time spent watching TV, hour/week, mean ± SD	2.4 ± 1.5	2.5 ± 1.5	2.4 ± 1.6	0.500
Fiber (grams/day), mean ± SD	24.4 ± 8.0	25.1 ± 8.0	24 ± 7.6	0.477
Number of chronic conditions, mean ± SD	0.7 ± 0.7	0.7 ± 0.7	0.7 ± 0.8	0.400
Number of medications per day, mean ± SD	1.7 ± 1.7	1.8 ± 1.8	1.7 ± 1.9	0.389
Hypertension, %	63.7	64.3	56.4	0.118 †
Diabetes mellitus, %	13	12.8	15.3	0.471 †
Hypercholesterolemia, %	70.8	72.1	73.6	0.643
BMI baseline, mean ± SD	28.1 ± 4	28.5 ± 4.4	28.6 ± 4.2	0.124

SD = Standard deviation. † Chi-Squared. The cutoff points for tertiles of the percentage of energy from ultra-processed food were: Tertile 1 (0–11.8), Tertile 2 (11.8–20.9), Tertile 3 (21–57.5) in men; Tertile 1 (0–13.9), Tertile 2 (14–23.8), Tertile 3 (23.9–66.7) in women.

**Table 2:** Association between the consumption of ultra-processed food expressed as a percentage of total energy (% energy) or as grams per day/weight and the risk of renal function decline after 6-year of follow-up (2008/10–2015) (*n* = 1312).

	T1 (Lowest) OR (95% CI)	T2 OR (95% CI)	T3 (Highest) OR (95% CI)	*p* Trend
Ultra-Processed Food Consumption (% Energy)				
*n*	438	438	436	
Cases	47	67	69	
Model 1	Ref.	1.63 (1.08–2.44)	1.75 (1.16–2.64)	0.008
Model 2	Ref.	1.56 (1.04–2.35)	1.69 (1.11–2.55)	0.014
Model 3	Ref.	1.56 (1.02–2.38)	1.74 (1.14–2.66)	0.026
Ultra-Processed Food Consumption (g/kg/Day)				
*n*	438	437	437	
Cases	55	61	67	
Model 1	Ref.	1.26 (0.84–1.89)	1.56 (1.03–2.35)	0.034
Model 2	Ref.	1.25 (0.84–1.88)	1.57 (1.04–2.38)	0.033
Model 3	Ref.	1.28 (0.85–1.95)	1.62 (1.06–2.49)	0.043

OR: Odds Ratio. CI: Confidence interval. Model 1: Logistic regression model adjusted for sex, age, and total energy intake. Model 2: As in Model 1 and additionally adjusted for education level (primary, secondary, university), smoking status (never, former, current smoker), former-drinker status (yes, no), physical activity (MET-hour/week), time spent watching TV (hour/week), and total fiber consumption (grams/day). Model 3: As in Model 2 and additionally adjusted for number of chronic conditions (continuous), number of medications used (continuous), hypertension (yes/no), and diabetes (yes/no), hypercholesterolemia (yes/no) and body mass index (continuous).

**Table 3:** Association between the consumption of ultra-processed food expressed as a percentage of total energy (% energy) and the risk of renal function decline after 6-year of follow-up (2008/10–2015) (*n* = 1312) according to morbidity and several cardiovascular risk factors.

	Ultra-Processed Food Consumption (% Energy)			
	T1 (Lowest)	T2	T3 (Highest)	*p* Trend
With at least one chronic condition				
*n*/cases	229/25	241/38	232/38	
OR (95% CI)	1 (Ref.)	1.49 (0.85–2.62)	1.5(0.84–2.68)	0.174
Without any chronic condition				
*n*/cases	209/22	197/29	204/31	
OR (95% CI)	1 (Ref.)	1.47 (0.78–2.76)	1.61 (0.86–3.03)	0.137
With hypertension				
*n*/cases	279/33	282/48	246/45	
OR (95% CI)	1 (Ref.)	1.54 (0.94–2.53)	1.65 (0.99–2.75)	0.055
Without hypertension				
*n*/cases	159/14	156/19	190/24	
OR (95% CI)	1 (Ref.)	1.49 (0.68–3.24)	1.52 (0.70–3.27)	0.305
With diabetes				
*n*/cases	57/8	56/14	67/22	
OR (95% CI)	1 (Ref.)	1.86 (0.62–5.6)	3.08 (1.08–8.75)	0.034
Without diabetes				
*n*/cases	381/39	382/53	369/47	
OR (95% CI)	1 (Ref.)	1.43 (0.91–2.25)	1.36 (0.85–2.19)	0.200
With hypercholesterolemia				
*n*/cases	310/34	316/47	321/53	
OR (95% CI)	1 (Ref.)	1.50 (0.92–2.46)	1.67 (1.03–2.73)	0.042
Without hypercholesterolemia				
*n*/cases	128/13	122/20	115/16	
OR (95% CI)	1 (Ref.)	1.63 (0.72–3.70)	1.38 (0.59–3.27)	0.474
With obesity (BMI ≥ 30 kg/m^2^)				
*n*/cases	125/17	141/27	128/18	
OR (95% CI)	1 (Ref.)	1.55 (0.76–3.14)	1.08 (0.50–2.32)	0.833
Without obesity (BMI < 30 kg/m^2^)				
*n*/cases	313/30	297/40	308/51	
OR (95% CI)	1 (Ref.)	1.49 (0.88–2.53)	1.90 (1.13–3.19)	0.015

All adjustments as in model 3. OR: Odds Ratio. CI: Confidence Interval. The considered chronic conditions were: chronic respiratory disease, coronary heart disease, stroke, heart failure, osteoarthritis, cancer, and depression requiring treatment. Hypertension: systolic blood pressure ≥140 mmHg or diastolic blood pressure ≥90 mmHg or antihypertensive treatment. Diabetes: fasting glucose ≥126 mg/dl or antidiabetic treatment. Hypercholesterolemia: total cholesterol ≥200 mg/dl or drug treatment. Obesity: BMI ≥ 30 kg/m^2^.
